# Astaxanthin Relieves Testicular Ischemia-Reperfusion Injury—Immunohistochemical and Biochemical Analyses

**DOI:** 10.3390/jcm11051284

**Published:** 2022-02-26

**Authors:** Marko Bašković, Dajana Krsnik, Marta Himelreich Perić, Ana Katušić Bojanac, Nino Sinčić, Zdenko Sonicki, Davor Ježek

**Affiliations:** 1 Scientific Centre of Excellence for Reproductive and Regenerative Medicine, School of Medicine, University of Zagreb, Šalata 3, 10000 Zagreb, Croatia; dajana.krsnik@mef.hr (D.K.); marta.himelreich@mef.hr (M.H.P.); ana.katusic@mef.hr (A.K.B.); nino.sincic@mef.hr (N.S.); zdenko.sonicki@snz.hr (Z.S.); davor.jezek@mef.hr (D.J.); 2 Department of Pediatric Urology, Children’s Hospital Zagreb, Ulica Vjekoslava Klaića 16, 10000 Zagreb, Croatia; 3 Department of Biology, School of Medicine, University of Zagreb, Šalata 3, 10000 Zagreb, Croatia; 4 Department of Medical Statistics, Epidemiology and Medical Informatics, School of Public Health Andrija Štampar, School of Medicine, University of Zagreb, Johna Davidsona Rockfellera 4, 10000 Zagreb, Croatia; 5 Department of Histology and Embryology, School of Medicine, University of Zagreb, Šalata 3, 10000 Zagreb, Croatia; 6 Department of Transfusion Medicine and Transplantation Biology, University Hospital Centre Zagreb, Kišpatićeva 12, 10000 Zagreb, Croatia

**Keywords:** astaxanthin, testicular torsion, acute scrotum, ischemia-reperfusion injury, antioxidants, carotenoids, apoptosis, infertility, rats

## Abstract

Testicular torsion potentially leads to acute scrotum and testicle loss, and requires prompt surgical intervention to restore testicular blood flow, despite the paradoxical negative effect of reperfusion. While no drug is yet approved for this condition, antioxidants are promising candidates. This study aimed to determine astaxanthin’s (ASX), a potent antioxidant, effect on rat testicular torsion−detorsion injury. Thirty-two prepubertal male Fischer rats were divided into four groups. Group 1 underwent sham surgery. In group 2, the right testis was twisted at 720° for 90 min. After 90 min of reperfusion, the testis was removed. ASX was administered intraperitoneally at the time of detorsion (group 3) and 45 min after detorsion (group 4). Quantification of caspase-3 positive cells and oxidative stress markers detection were determined immunohistochemically, while the malondialdehyde (MDA) value, superoxide dismutase (SOD), and glutathione peroxidase (GPx) activities were determined by colorimetric assays. The number of apoptotic caspase-3 positive cells and the MDA value were lower in group 4 compared to group 2. A significant increase in the SOD and GPx activity was observed in group 4 compared to groups 2 and 3. We conclude that ASX has a favorable effect on testicular ischemia-reperfusion injury in rats.

## 1. Introduction

Testicular torsion is a condition of acute scrotum, starting with the rotation of the testis around a longitudinal axis by at least 180 degrees, and followed by an interruption of circulation inside the organ. Despite the possibility of manual detorsion, surgery is usually required and should be performed as soon as possible after the onset of symptoms. If not recognized in time, it can result in ischemic injuries and testicular loss, but if the operation is performed within 6 h, most testicles can be saved [1,2,3]. The incidence of testicular torsion is 1 in 4000 males younger than 25 years, while the prevalence of testicular torsion out of a total of all acute scrotal conditions is 25–50% [4,5,6]. It can occur at any age, but most often shows a bimodal distribution, i.e., it most often occurs in infants and boys at puberty [7,8], usually occurring after some stimulus event (e.g., trauma or increased physical activity) or spontaneously [9]. Clinical features of testicular torsion include the acute onset of moderate to severe testicular pain with the possibility of the presence of redness and swelling with a negative cremaster reflex during physical examination. Nausea, vomiting, and diffuse pain in the lower abdomen may be associated with this condition. The classic clinical finding is an asymmetrically (transversely) highly laid testis [10,11].

Ischemia-reperfusion injury (IRI) exacerbates cell dysfunction observed after restoring blood flow in previously ischemic tissues. Hence, reperfusion paradoxically causes further damage, endangering the organ vitality and function despite the necessity for blood flow restoration. Reperfusion injury is a multifactorial process that results in tissue destruction [12]. During reperfusion, the influx of oxygen leads to the degradation of hypoxanthine to uric acid by enzyme xanthine oxidase. This reaction releases highly reactive anion superoxide (O^2−^), which is then converted to hydrogen peroxide (H_2_O_2_) and hydroxyl radical (OH·). The main unwanted consequence of the production of hydroxyl radicals is membrane lipid peroxidation. Lipid peroxidation causes the systemic release of proinflammatory eicosanoids, disruption of cell permeability, and ultimately cell death [13,14,15,16]. The increase in the concentration of free oxygen radicals most often occurs if the mechanisms in charge of removing them become insufficient. This upsets the balance between prooxidants and antioxidants, favoring prooxidants (a state of oxidative stress). Cell damage is reversible up to one point, but with intense and prolonged stress, the cell is subject to irreversible damage [17]. While low concentrations of free oxygen radicals induce apoptosis, high ones result in necrosis. Cysteine proteases that form a large family of enzymes known as caspases cause most cell morphological changes [18,19].

Antioxidants are molecules that, by inhibiting the oxidation of other molecules, defend the body’s system against potential damage by free oxygen radicals [20]. In recent decades, interest in natural sources of antioxidants has risen sharply. Algae constitute a significant source of molecules with an antioxidant activity, as they often grow in extreme environmental conditions, resulting in the production of large numbers of free oxygen radicals. To ameliorate their effect, algae create various secondary metabolites with antioxidant activities such as phycobilins, polyphenols, carotenoids, and vitamins [21].

The carotenoid pigment astaxanthin (ASX) (C_40_H_52_O_4_), found in the microalgae *Haematococcus pluvialis*, has anti-inflammatory, immunomodulatory, and antioxidant effects [22]. ASX is also found in salmon, shrimp, and crabs, giving them a specific shade of red [23]. Compared to other carotenoids such as beta-carotene, zeaxanthin, and canthaxanthin, ASX shows higher levels of antioxidant activity [24]. The antioxidant activity of ASX is ten times higher than zeaxanthin, canthaxanthin, β-carotene, and lutein, and 100 times higher than α-tocopherol [25].

For these benefits, we decided to investigate the ASX’s effect on testicular IRI. We previously published comprehensive histological results showing that ASX has a protective effect [26,27]. Still, only a multimodal approach can strengthen the hypothesis, we showed the results of immunohistochemical and biochemical analyses in this study. There is no drug in clinical practice that can be given to patients with torsion−detorsion testicular injury to date. We believe this study gives a new insight into the possible treatment of this urgent condition and its consequences (subfertility and infertility).

## 2. Materials and Methods

### 2.1. Animals

The study was performed on 32 male Fischer rats (weight 160–210 g, 35 days old) of prepubertal age. The animals were housed under the conditions following good laboratory practice (GLP), which included a temperature of 20–24 °C, relative humidity 55% +/− 10%, controlled lighting, and light dark cycle of 12 h/12 h. The noise level did not exceed 60 dB.

### 2.2. Ethics Approvals

The research was approved by the School of Medicine, University of Zagreb (classification; 641-01/19-02/01/registry number; 380-59-10106-19-111/162) and the Croatian National Ethics Committee (EP 217/2019). The 3R principles were used—“reduction”, “refinement”, and “replacement”—and the concept of five freedoms was respected.

### 2.3. Experimental Groups and Surgical Procedure

Rats were randomly divided into four groups with eight individuals in each group, namely: sham-operated (S) group, torsion−detorsion (T/D) group, and torsion−detorsion + astaxanthin (T/D + ASX) groups.

Group 1 (S) underwent sham surgery. After the intraperitoneal injection of anesthetic, an incision was made in the right inguinal region, to pull out the ipsilateral testis, which was immediately returned to its natural position and the skin sutured. After suture removal, orchidectomy was performed after 3 h. In group 2 (T for 90 min/D for 90 min), the ipsilateral testis was twisted around its axis by 720° in a clockwise direction. It was fixed in that position for 90 min. After 90 min, detorsion was performed. The skin was sutured twice (0 min and 90 min). Orchidectomy was performed 90 min from the moment of detorsion. At the time of detorsion, group 3 (T for 90 min/D for 90 min + ASX at the time of detorsion) was administered pure ASX intraperitoneally (75 mg/kg, Sigma-Aldrich^®^, St. Louis, MO, USA, from *Blakeslea trispora*). In group 4 (T for 90 min/D for 90 min + ASX 45 min from the moment of detorsion) ASX was administered 45 min after detorsion.

All surgical procedures were performed under aseptic conditions. After shaving the right inguinoscrotal region, washing with chlorhexidine gluconate (PLIVA^®^sept, Pliva d.o.o., Zagreb, Croatia), and drying, the area was treated with a povidone-iodine solution (Betadine^®^10%, Alkaloid, Skopje, North Macedonia). In the midline of the scrotum, an incision was made. Upon opening the tunica vaginalis, the testis was twisted manually around its axis by 720° in a clockwise direction. The testis was fixed to the inner wall of the scrotum with a monofilament polyglactin suture 6/0 (Vicryl; Ethicon Inc., Johnson and Johnson Co., Somerville, NJ, USA). By removing the suture, the right testicle was manually returned to its natural position. The skin of the scrotum was also sutured with a monofilament polyglactin suture 6/0. All surgical procedures were performed under general anesthesia induced by intraperitoneal injection of ketamine (90 mg/kg) and xylazine (10 mg/kg). The animals were constantly monitored. In case of movement, twitching, or other signs of awakening, intraperitoneal anesthesia was supplemented in a smaller dose. No animals died during the experiment. After orchidectomy, the rats were euthanized using the T-61 solution (1 mL/kg) iv. (Intervet International GmbH^®^, Unterschleißheim, Germany).

### 2.4. Immunohistochemical Method and Analysis

The immunohistochemical method was used to evaluate the cell damage exhibited by apoptosis and oxidative stress in the testicular tubules after treatment. Anti-cleaved caspase-3 antibody (1:100, #9664, Cell Signaling Technology^®^, Danvers, MA, USA) was used as an apoptotic marker, while anti-8-oxo-2′-deoxyguanosine (anti 8-OHdG), anti-nitrotyrosine (anti-NT) (1:300, sc-66036 and 1:100, sc-32757, respectively, Santa Cruz Biotechnology, Inc., Dallas, TX, USA) and anti-4-hydroxy-2-nonenal (anti-HNE) antibodies (MAB3249 R&D Systems, Inc., Minneapolis, MN, USA) were used as oxidative stress markers. After overnight incubation with primary antibody at 4 °C, the sections were treated with appropriate secondary antibodies. The signal was visualized using 3,3′-diaminobenzidine-tetrahydrochloride (DAB) and hematoxylin for counterstaining. Positive control tissues were used, as recommended by the manufacturer of the antibodies, while the negative controls were gained by omitting the primary antibody in the buffer. To detect caspase-3 positive cells as clearly as possible, the “invert” option was used in the ImageJ^®^ software (software package developed by the National Institutes of Health). The number of caspase-3-positive cells was determined by counting 100 random seminiferous tubules (apoptotic index) (x400). Caspase-3 positive cells were counted by visual observation from two independent researchers. If the numbers differed, the opinion of a third researcher was sought. Data are expressed as the mean of caspase-3-positive cells per 100 seminiferous tubules. Descriptive analysis of antibodies against oxidative stress markers was performed to evaluate the histological localization on six samples per group.

### 2.5. Biochemical Analysis

The values of malondialdehyde (MDA) and enzymatic antioxidants (superoxide dismutase (SOD) and glutathione peroxidase (GPx)) were determined by colorimetric assays using the testicular tissue homogenates as the samples. The MDA Assay Kit (MAK085, Sigma-Aldrich^®^, St. Louis, MO, USA) was used to measure lipid peroxidation. According to the manufacturer’s protocol, the MDA in the homogenized sample makes a complex with thiobarbituric acid (TBA), which could be quantified colorimetrically (532 nm) on a spectrophotometer (Tecan Spark, Tecan, Life Sciences). The SOD activity was analyzed with the colorimetric SOD determination kit (19160, Sigma-Aldrich^®^, St. Louis, MO, USA). Tetrazolium salt was used as a substrate (WST), which produces a water-soluble formazan dye after reduction with a superoxide anion. The rate of WST reduction was linearly related to the xanthine oxidase (XO) activity, but concomitantly inhibited by SOD. IC50 (50% SOD inhibition activity) was determined by the colorimetric method. As the absorption at 440 nm is proportional to the amount of superoxide anion, the activity of SOD as an inhibitory activity was quantified by measuring the decrease in color development at 440 nm. The GPx Assay Kit (353919; Sigma-Aldrich^®^, St. Louis, MO, USA) measured GPx activity. The main reaction catalyzed by GPx is 2GSH + H_2_O_2_ → GS–SG + 2H_2_O, where GSH is the reduced monomeric glutathione and GS–SG glutathione disulfide. The mechanism involves the oxidation of selenol in the selenocysteine residue via hydrogen peroxide. Glutathione reductase then reduces oxidized glutathione and completes the following cycle: GS–SG + NADPH + H+ → 2GSH + NADP+. Oxidation of NADPH to NADP+ was accompanied by a decrease in absorption to 340 nm. Under conditions where GPx activity is limited, the rate of decrease in A₃₄₀ is directly proportional to the GPx activity in the sample. The amount of NADPH in the reaction mixture was determined kinetically by reading the ΔA₃₄₀ absorbance value at 340 nm at 1 min intervals over the 7 min time frame.

### 2.6. Statistical Analysis

Microsoft Excel^®^ software program (XLSTAT^®^) for Windows, version 2020.5.1 (Microsoft Corporation, Redmond, DC, USA), was used to analyze the experimental data. Before the study, power analysis was performed where a sample of four groups of eight animals was shown to be required (for α = 0.05, power = 95% and effect ≥ 0.9) in order to obtain high-quality data. The Shapiro−Wilk test was used for the normal distribution assessment of collected measurements mainly presented by the interquartile range (median). Differences between groups were analyzed by the nonparametric Kruskal−Wallis test. The data were presented as follows; chi-square (χ2) = observed value (critical value), degrees of freedom (DF), and *p*-value. The Mann−Whitney U test with Bonferroni correction was used for the pairwise comparisons. A significance level of 0.05 was used.

## 3. Results

### 3.1. Caspase-3 Positive Cells Quantification

**Figure 1:**
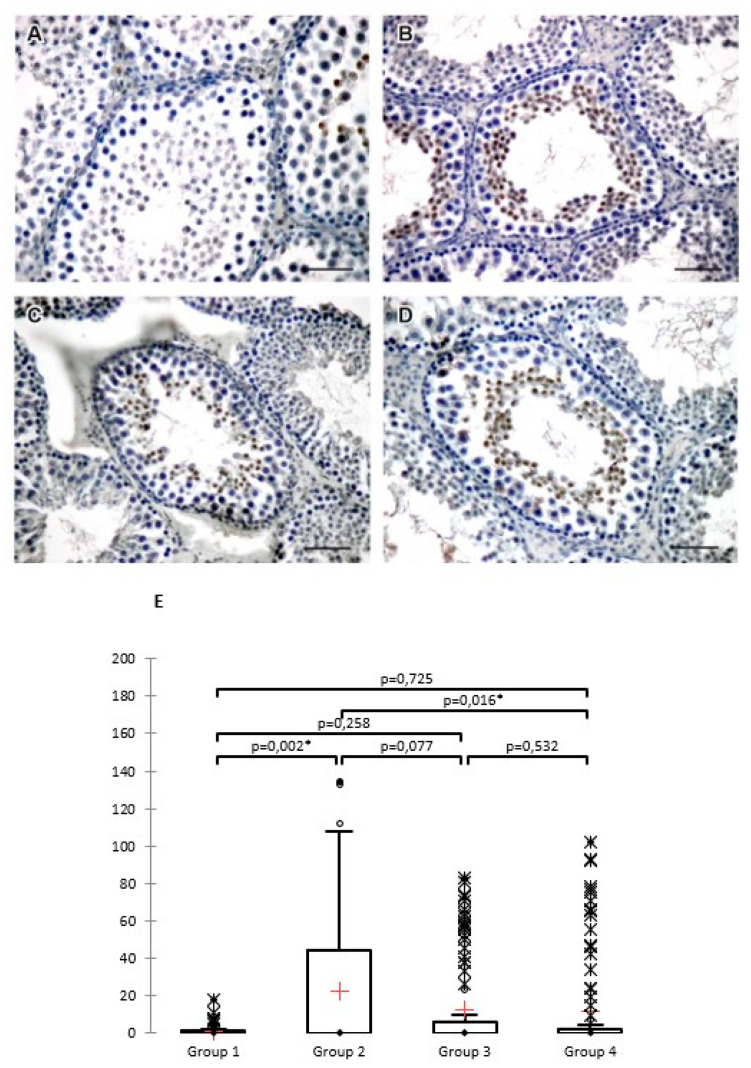
Caspase-3 positive cells on representative, randomly selected cross-sections on which the measurements were performed: (**A**) group 1, (**B**) group 2, (**C**) group 3, and (**D**) group 4. DAB, hematoxylin counterstain, scale bar 50 µm. (**E**) Box plots for caspase-3 positive cells (data are presented as mean ± SD). A Kruskal−Wallis test revealed a statistically significant difference in the number of caspase-3 positive cells between the different groups (at a significance level of 5%); (χ^2^ = 10.441 (7.815), DF = 3, *p* = 0.015), * *p* < 0.05.

The number of caspase-3-positive cells was statistically significantly lower (*p* = 0.016) in group 4, in which ASX was administered 45 min from the time of detorsion (mean = 11.84) compared to the untreated torsion−detorsion group 2 (mean = 22,700). Compared to group 2, group 3, in which ASX was administered at the time of detorsion, recorded a far lower mean (mean = 12.50), but there was no statistically significant difference (*p* = 0.077; Table S1 and Figure 1).

### 3.2. Histological Assessment of Oxidative Stress

**Figure 2:**
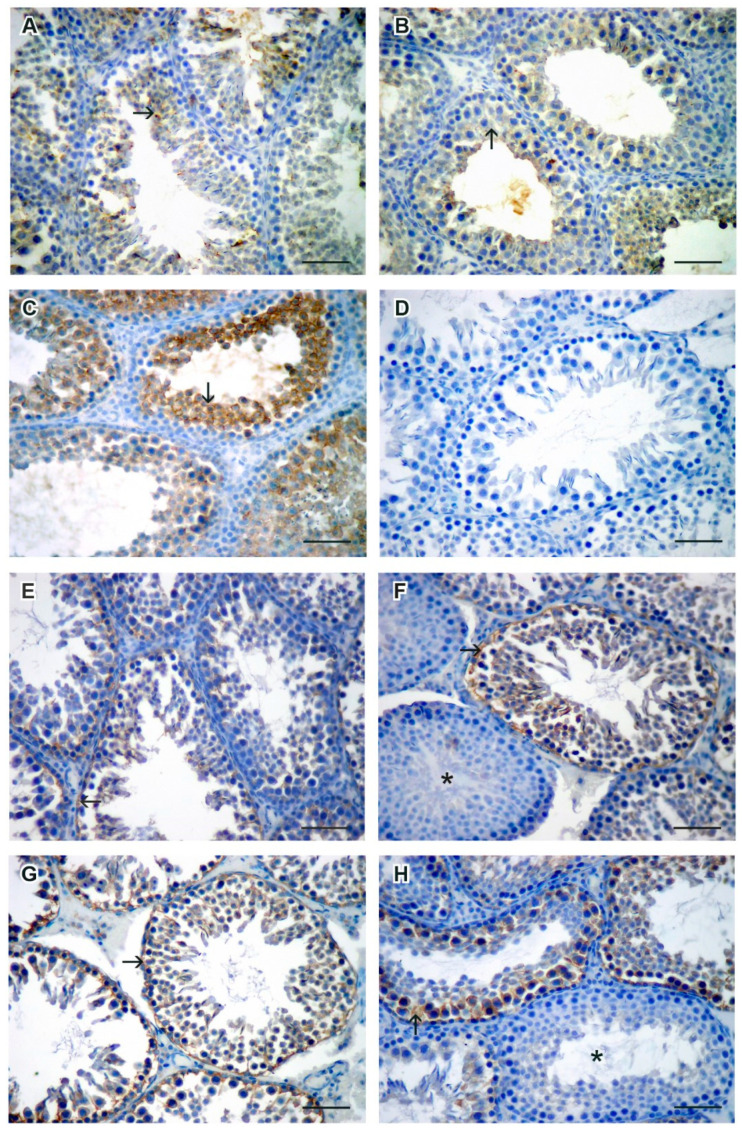
Representative images of HNE (**A**–**D**) and 8-OHdG (**E**–**H**) expression (→) in the rat testes of groups 1 (**A**,**E**), 2 (**B**,**F**), 3 (**C**,**G**), and 4 (**D**,**H**). Note the difference in the expression on neighboring tubules in images F and H (*- nonaffected tubule). DAB, hematoxylin counterstain, scale bar 50 µm.

8-hydroxy-2’deoxyguanosine (8-OHdG), the marker of oxidative DNA damage, was found in most tubules of all groups, although it was more intensely stained in group 3, and was without visible tubules with no affection in the same group. The signal was cytoplasmic, limited to the basal layer of the Sertoli cells and spermatogonia, near the tubular wall. In all groups except group 3, there were completely unaffected tubules next to those with a damaged histological appearance (Figure 2G).

4-hydroxy-2-nonenal (HNE), the marker of lipid peroxidation, showed the strongest staining intensity in group 3, affecting the entire height of the seminiferous epithelium (Figure 2C). Group 4 had a staining signal similar to the negative control (Figure 2D).

Nitrotyrosine staining showed no positive signal in the specimens, while the positive control was stained as expected.

### 3.3. Values of Malondialdehyde (MDA)

**Figure 3:**
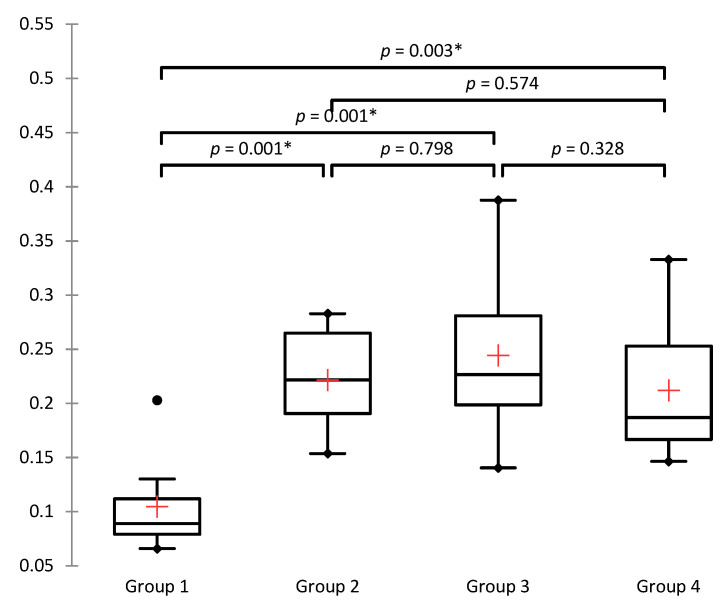
Box plots for malondialdehyde (nmol/μg). The Kruskal−Wallis test shows a statistically significant difference in the observed parameters between different groups (at a significance level of 5%); (χ^2^ = 14.395 (7.815), DF = 3, *p* = 0.002), * *p* < 0.05.

Malondialdehyde values decreased in the group in which ASX was administered 45 min from the moment of detorsion (Mdn = 0.187) compared to the untreated torsion−detorsion group (Mdn = 0.222), but the difference was not statistically significant (*p* = 0.574). The median values between group 2 (Mdn = 0.222) and group 3 (Mdn = 0.227) were almost identical (*p* = 0.798). The MDA values in group 2 in relation to the negative control group increased significantly (*p* = 0.001) (Table S2 and Figure 3).

### 3.4. Values of Superoxide Dismutase (SOD)

**Figure 4:**
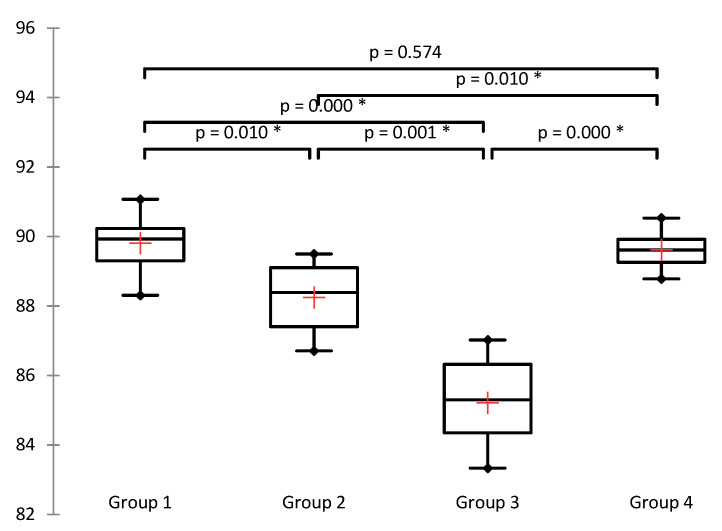
Box plots for SOD activity (inhibition rate %). The Kruskal−Wallis test shows a statistically significant difference in the observed parameter between different groups (at a significance level of 5%); (χ^2^ = 22.023 (7.815), DF = 3, *p* < 0.0001), * *p* < 0.05.

Following the results, a statistically significant increase in the enzyme activity of superoxide dismutase (SOD) was observed in group 4, in which ASX was administered 45 min from the moment of detorsion (Mdn = 89.61) compared to untreated torsion−detorsion group 2 (Mdn = 88.39) (*p* = 0.01) and group 3, in which ASX was administered at the time of detorsion (Mdn = 85.30) (*p* = 0.000). It is interesting to note a statistically significant decrease in the enzyme activity of SOD in group 3 compared to group 2 (*p* = 0.001; Table S3 and Figure 4).

### 3.5. Values of Glutathione Peroxidase (GPx)

**Figure 5:**
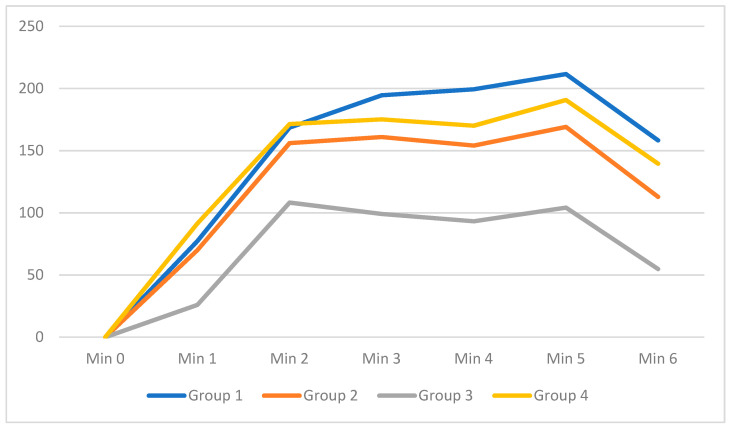
Linear graph of GPx activity (nmol/min/mL) over time from the first to the sixth minute.

The Kruskal−Wallis test showed a statistically significant difference in the observed parameters between different groups (at a significance level of 5%); first minute (χ^2^ = 17.020 (7.815), DF = 3, *p* = 0.001), second minute (χ^2^ = 13.497 (7.815), DF = 3, *p* = 0.004), third minute (χ^2^ = 14.838 (7.815), DF = 3, *p* = 0.002), fourth minute (χ^2^ = 17.701 (7.815), DF = 3, *p* = 0.001), fifth minute (χ2 = 18.637 (7.815), DF = 3, *p* = 0.000), sixth minute (χ^2^ = 19.431 (7.815), DF = 3, *p* = 0.000) (Table S4, Figures S1–S6, and Figure 5).

## 4. Discussion

The results of this study showed that ASX has a favorable effect on ischemia-reperfusion testicular injury (IRI) in rats. In the immunohistochemical part of the study, we found that there was a decrease in the number of apoptotic caspase-3 positive cells in the ASX groups compared to the torsion−detorsion group in which ASX was not applied (group 2) and statistically significant when ASX was applied 45 min from the moment of detorsion (group 4). Furthermore, biochemical studies showed a decrease in malondialdehyde values and an increase in the enzyme activity of superoxide dismutase and glutathione peroxidase in group 4. Although the malondialdehyde values did not decrease significantly, the observed median decreased. The superoxide dismutase enzyme activity increased significantly in group 4 compared to groups 2 and 3. The same pattern of results was observed for the glutathione peroxidase enzyme activity in the first six minutes. It is also interesting to note statistically significant decreases in group 3 compared to group 2 in the superoxide dismutase and glutathione peroxidase enzyme activity. We expected the ameliorating effect of ASX on the torsion to be stronger in group 3 compared to group 4 because, in group 3, ASX was applied concomitantly with detorsion. Still, the results of all measured variables were closer to the negative control in group 4. This may be due to the sluggish return of the blood flow, which can limit vascular capacity to deliver appropriate doses of antioxidants to the testes during the immediate post-torsion period. By prolonging the duration of torsion, the return of blood after detorsion is slower. It is important to note that the first 60–90 min after the initial reperfusion is a critical time, for a toxic outbreak of free oxygen radicals [28].

Several studies have reported a cytoplasmic 8-OHdG expression [29,30,31], in concordance with our study and reports of 8-OHdG accumulating in mitochondrial DNA, although it is known to be found in the nuclei [32]. The finding of unaffected tubules shows that some tubules avoid ischemia and necrosis if the torsion persists, and these findings are in concordance with the expression pattern of oxidative stress markers 8-OHdG and NT. The strongest signal, being in group 3, treated with ASX at the time of the torsion, may be due to the induction of oxidative stress markers expression as signaling molecules in different cascades of tissue repair [33] or the edema, which prevents the transport of ASX to the testicular tissues.

This study focused on the acute effect and acute changes after IRI, but in everyday clinical practice, the average time from torsion to surgery often exceeds 90 min. To mimic real-life settings, the study would benefit from extending the time from torsion to surgery. Prolonging the time from torsion to reperfusion can be considered in future studies. ASX was administered intraperitoneally, as this route of administration was most appropriate for this model. We are aware that oral and intravenous routes of administration are more applicable for human administration, but as more detailed pharmacokinetic and pharmacodynamic studies are ongoing, we believe that intraperitoneal administration is more than satisfactory for testing ASX as a potentially potent antioxidant in preventing IRI. We opted for a dose of 75 mg/kg, but believe that in future studies, the dose may be reduced to keep the dose within the range currently recommended for use in humans, even though no adverse effects have been found in recent toxicological studies and at much higher doses. Next, we show that the slow return of blood could influence the effectiveness of the applied antioxidant, but we also point to the more beneficial effect of ASX when applied 45 min after detorsion than at the time of detorsion. Additional experimental groups should be included in the study to determine the optimal time for the ASX administration. Each group would be given ASX at a successively different time from the moment of detorsion. For example, regarding the already known harmful effect of IRI of the ipsilateral on the contralateral testis, one would also have to explore the ASX potential in ameliorating this effect.

The effects of ASX on testicular torsion have not been investigated prior to our study, although the effects regarding its precursor lycopene have been. Hekimoglu et al. [34] investigated changes after one-hour vascular clamp ischemia, and after three-hour and twenty-four-hour reperfusions. Analogous to our results in the previous study [26,27], the group receiving lycopene statistically significantly improved the Johnsen score in the testis, compared to the group in which only torsion−detorsion was performed. Analogous to the results presented here, Hekimoglu et al. showed that the values of GPx activity in the lycopene group approached the values of the sham group, demonstrating a protective effect. Malondialdehyde values, analogous to our results, were similar in all groups, with no statistically significant difference, but the mean values were lower in the groups in which lycopene was administered, supporting its protective effect. We must point out that in preclinical studies, ischemia should be performed by manual torsion rather than by vascular ligation with a vascular clamp. The torsion initially clogs veins but not arteries, and thus causes partial ischemia in the early torsion period. Güzel et al. [35] investigated the effect of intraperitoneal lycopene administration. In their model, torsion of 720° lasted for two hours, after which lycopene was given for three and ten days at a dose of 20 mg/kg/day. The mean seminiferous tubule diameter and Johnsen score were higher in the group receiving lycopene for three days intraperitoneally compared with the group without lycopene. In addition, in the groups in which lycopene was administered, a smaller number of apoptotic cells were observed by the TUNEL method, while the MDA values decreased in both groups that received lycopene for three and ten days. The SOD values did not show this tendency, while in our study, in group 4, the SOD values showed a statistically significant increase compared to group 2. From this, it undoubtedly follows that ASX has a far more potent effect than its precursor, but we must keep in mind that lycopene was administered at a dose of 20 mg/kg/day, while we administered ASX at a dose of 75 mg/kg. Compared to the studies mentioned earlier [34,35], we must note that we used prepubertal rats in our study due to the well-known fact that testicular torsion in humans occurs primarily in adolescence and preadolescence [36]. It is also important to note that Hekimoglu et al., compared to Güzel et al., gave lycopene by gavage. The route of administration of the potential drugs is of great importance, as some studies have shown limitations after oral administration, such as low stability, bioavailability, and bio-efficiency with ASX, revealing the need for new biomaterials acting as carriers in vivo [37]. Given the results of previous research as well as our research, it would certainly be interesting to investigate the possible beneficial effects of other compounds from the biosynthetic pathway of ASX, such as β-carotene, zeaxanthin, canthaxanthin, and violaxanthin [38].

Although it has been known for centuries that certain natural derivatives (exogenous factors) have beneficial effects on human health and the male reproductive system, it is only in recent decades that they have become increasingly important. Many are already registered as dietary supplement and are presented on the pharmaceutical market as supplements [21,39,40]. Currently, the main carotenoids of market interest are β-carotene, ASX, lutein, zeaxanthin, lycopene, and canthaxanthin. ASX and β-carotene are the two most well-known carotenoids in the global market and make up almost half of the carotenoid market (according to *Business Communications Company*, 2015). The total carotenoid market in 2019 was $1.8 billion, and β-carotene, lutein, and ASX accounted for more than 60% of the market share [22,41,42,43]. The beneficial effects of ASX are reflected in several studies. Otsuka et al. [44] concluded that the use of ASX could effectively protect against neurodegeneration during ischemic retinopathy. ASX has shown optimistic results in IRI of the liver and muscles [45,46], while the myocardium had a beneficial effect regarding IRI from disodium disuccinate ASX [47]. The preservation of renal function has been observed in a mouse kidney model [48]. While Tripathi and Jena [49] observed a protective effect on the germ cells protector in cyclophosphamide-treated mice, the positive effect of ASX on steroidogenesis in Leydig cells was described by Wang et al. [50].

Within the European Union, ASX from natural sources is currently sold in daily doses of up to 12 mg and is approved by national authorities worldwide in daily doses of up to 24 mg. Critical determinants of ASX’s ability to properly integrate into its molecular environment to increase its activity are structural features such as size, shape, and polarity [51]. To date, studies in more than 2000 participants have found no significant toxicity at any dose for natural ASX, which has shown an excellent clinical safety profile at short-term (up to 100 mg) and long-term daily doses (8 to 12 mg) [52]. In rats, safety was assessed by the daily oral administration of ASX-rich biomass at concentrations up to 500 mg/kg/day for 90 days, or synthetic ASX ranging between 880 and 1240 mg/kg/day for 13 weeks [53,54]. Katsumata et al. investigated a subchronic toxicity of daily administration of natural ASX by oral gavage at doses up to 1000 mg/kg/day for 13 weeks. The only observed result was the excretion of dark red color feces [55]. Given these results and the current knowledge, it is unlikely that there will be an obstacle to recommending higher than current doses for human use in the future.

Given the potential ethical issues and research length, to date, no clinical studies have been conducted on the effect of ASX on testicular IRI in humans. The effects of ASX on humans are being explored, showing its beneficial effect on the human body (e.g., ASX inhibits LDL oxidation and increases HDL levels, modulates the immune response, protects against UV radiation, is used in anti-aging treatments, inhibits proliferation of human gastric cancer cell lines, has genoprotective properties) [56,57,58,59,60,61,62]. As for male infertility, Comhaire et al. [63] observed positive effects on sperm parameters and fertility. Research on the pharmacokinetics and pharmacodynamics of ASX has not been completed, so we will have to wait for the optimal administration route determination [64,65]. The existence of the blood−testis barrier, as well as its changes due to ischemia during torsion, should not be overlooked [66].

## 5. Conclusions

Our study promotes ASX treatment on testicular ischemia-reperfusion injury. Given the rapid growth of research in the field of antioxidants and testicular ischemia-reperfusion injury, we believe that one day the powerful antioxidants, especially ASX, will be applicable in clinical settings, given that, to date, there is no cure given to patients.

## Data Availability

The data that support the findings of this study are available upon request from the corresponding author.

## Supplementary Materials

The following are available online at https://www.mdpi.com/article/10.3390/jcm11051284/s1, Figure S1: Box plots for for GPx activity in the 1st minute, Figure S2: Box plots for for GPx activity in the 2nd minute, Figure S3: Box plots for for GPx activity in the 3rd minute, Figure S4: Box plots for for GPx activity in the 4th minute, Figure S5: Box plots for for GPx activity in the 5th minute, Figure S6: Box plots for for GPx activity in the 6th minute, Table S1: Means, standard deviations, medians, Q1, Q3, and interquartile ranges by groups for caspase-3 positive cells, Table S2: Median, Q1, Q3, and interquartile range values by groups for MDA, Table S3: Median, Q1, Q3, and interquartile range values by groups for SOD, Table S4: Median, Q1, Q3, and interquartile ranges by groups for GPx activity in the 1st, 2nd, 3rd, 4th, 5th, and 6th minutes.
