# Supplementary material for: Astaxanthin Relieves Testicular Ischemia-Reperfusion Injury—Immunohistochemical and Biochemical Analyses
Source: J Clin Med. 2022 Feb 26;11(5):1284. doi: 10.3390/jcm11051284 (PMC8911179; doi:10.3390/jcm11051284)
Supplement: Supplementary file 1 [file jcm-11-01284-s001.zip › jcm-1594945-supplementary.pdf]

Supplementary

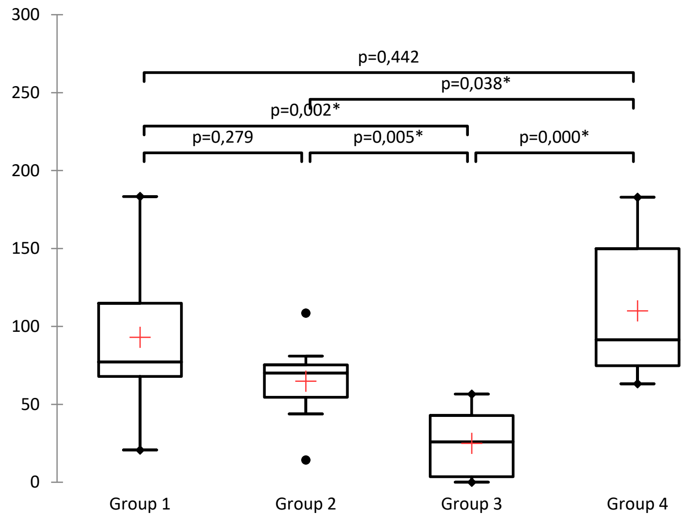

Figure S1. Box plots for for GPx activity in the 1st minute. There was a statistically significant increase in the enzymatic activity of glutathione peroxidase (GPx) in the first minute, in the group in which astaxanthin was administered 45 minutes from the moment of detorsion (Mdn = 91.43) compared to the untreated torsion-detorsion group (Mdn = 70.04) and the group in which astaxanthin was administered at the time of detorsion (Mdn = 25.98) [p (group 2/4) = 0.038, p (group 3/4) = 0.000]. It is also interesting to note a statistically significant decrease in GPx activity in group 3 compared to group 2 (p = 0.005).

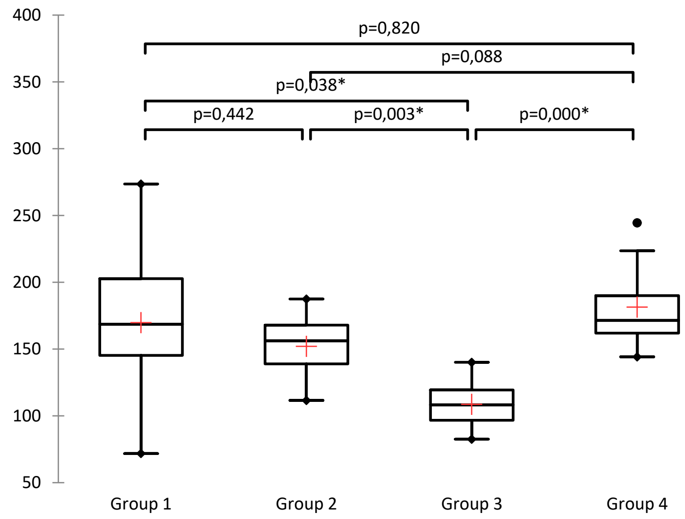

Figure S2. Box plots for for GPx activity in the 2nd minute. An increase in the enzymatic activity of glutathione peroxidase (GPx) was observed in the second minute, in the group in which astaxanthin was

administered 45 minutes from the moment of detorsion (Mdn = 171.41) compared to the untreated torsion-detorsion group (Mdn = 156.13) and the group in which astaxanthin was administered at the time of detorsion (Mdn = 108.21) [p (group 2/4) = 0.088, p (group 3/4) = 0.000]. It is also interesting to note a statistically significant decrease in GPx activity in group 3 compared to group 2 (p = 0.003).

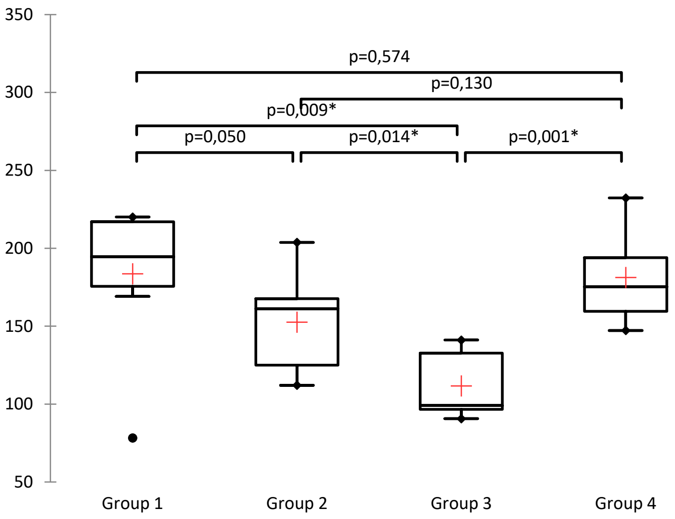

Figure S3. Box plots for for GPx activity in the 3rd minute. An increase in the enzymatic activity of glutathione peroxidase (GPx) was observed in the third minute, in the group in which astaxanthin was administered 45 minutes from the moment of detorsion (Mdn = 175.23) compared to the untreated torsion-detorsion group (Mdn = 161.22) and the group in which astaxanthin was administered at the time of detorsion (Mdn = 99.08) [p (group 2/4) = 0.130, p (group 3/4) = 0.001]. It is also interesting to note a statistically significant decrease in GPx activity in group 3 compared to group 2 (p = 0.014).

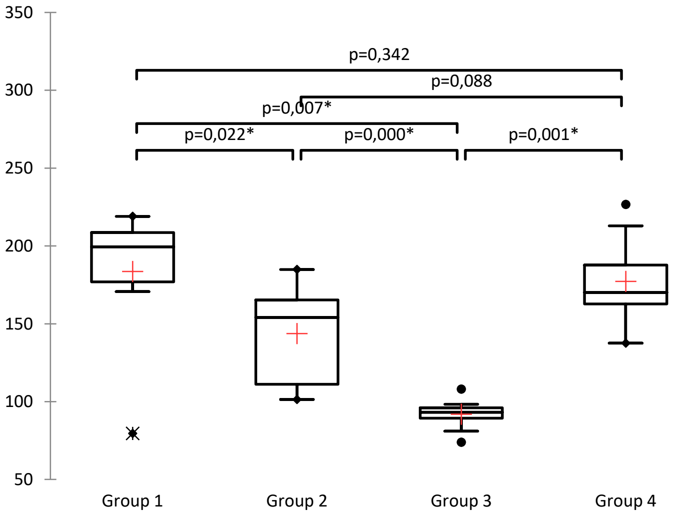

Figure S4. Box plots for for GPx activity in the 4th minute. An increase in the enzymatic activity of glutathione peroxidase (GPx) was observed in the fourth minute, in the group in which astaxanthin was administered 45 minutes from the moment of detorsion (Mdn = 170.13) compared to the untreated torsion-detorsion group (Mdn = 154.09) and the group in which astaxanthin was administered at the time of detorsion (Mdn = 93.22) [p

(group 2/4) = 0.088, p (group 3/4) = 0.001]. It is also interesting to note a statistically significant decrease in GPx activity in group 3 compared to group 2 (p = 0.000).

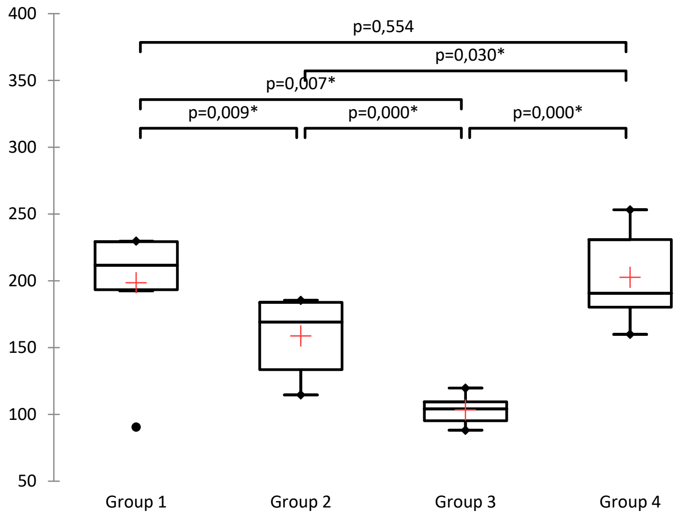

Figure S5. Box plots for for GPx activity in the 5th minute. There was a statistically significant increase in the enzymatic activity of glutathione peroxidase (GPx) in the fifth minute, in the group in which astaxanthin was administered 45 minutes from the moment of detorsion (Mdn = 190.76) compared to the untreated torsion-detorsion group (Mdn = 169.12) and the group in which astaxanthin was administered at the time of detorsion (Mdn = 104.17) [p (group 2/4) = 0.03, p (group 3/4) = 0.000]. It is also interesting to note a statistically significant decrease in GPx activity in group 3 compared to group 2 (p = 0.000).

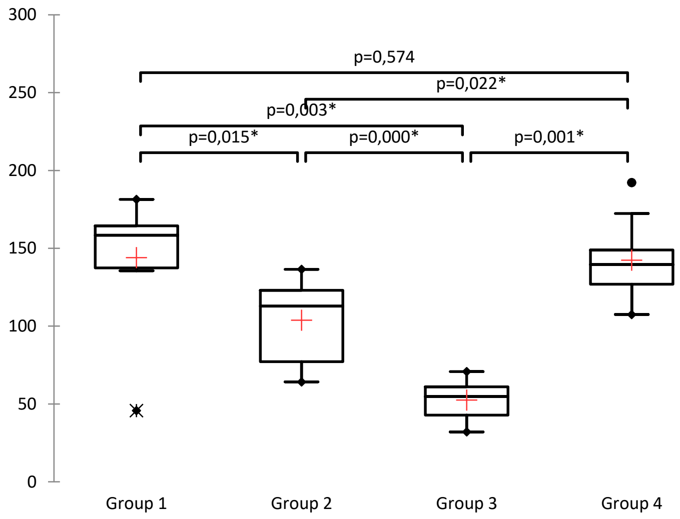

Figure S6. Box plots for for GPx activity in the 6th minute. There was a statistically significant increase in the enzymatic activity of glutathione peroxidase (GPx) in the sixth minute, in the group in which astaxanthin was administered 45 minutes from the moment of detorsion (Mdn = 139.57) compared to the untreated torsion-detorsion group (Mdn = 112.83) and the group in which astaxanthin was administered at the time of detorsion

(Mdn = 54.76) [p (group 2/4) = 0.022, p (group 3/4) = 0.001]. It is also interesting to note a statistically significant decrease in GPx enzyme activity in group 3 compared to group 2 (p = 0.000).

Table S1. Means, standard deviations, medians, Q1, Q3, and interquartile ranges by groups for caspase-3 positive cells

Group	Mean	SD	Mdn	Q1	Q3	IQR
**1**	1,130	2,677	0,000	0,000	1,000	1,000
**2**	22,700	35,218	0,000	0,000	44,500	44,500
**3**	12,500	23,976	0,000	0,000	6,000	6,000
**4**	11,840	25,343	0,000	0,000	2,000	2,000

SD, standard deviation; Mdn, median; Q1, quartile 1; Q3, quartile 3; IQR, interquartile range

Table S2. Median, Q1, Q3, and interquartile range values by groups for malondialdehyde

Group	Mdn	Q1	Q3	IQR
**1**	0,089	0,079	0,112	0,033
**2**	0,222	0,191	0,265	0,074
**3**	0,227	0,199	0,281	0,082
**4**	0,187	0,167	0,253	0,086

Mdn, median; Q1, quartile 1; Q3, quartile 3; IQR, interquartile range

Table S3. Median, Q1, Q3, and interquartile range values by groups for superoxide dismutase

Group	Mdn	Q1	Q3	IQR
**1**	89,929	89,300	90,231	0,931
**2**	88,392	87,408	89,103	1,695
**3**	85,299	84,350	86,324	1,974
**4**	89,611	89,257	89,922	0,665

Mdn, median; Q1, quartile 1; Q3, quartile 3; IQR, interquartile range

Table S4. Median, Q1, Q3, and interquartile ranges by groups for GPx activity in the 1st, 2nd, 3rd, 4th, 5th, and 6th minutes

GPx activity	Group	Mdn	Q1	Q3	IQR
**in the first minute**	**1**	77,172	67,875	114,866	46,991
	**2**	70,040	54,504	75,261	20,757
	**3**	25,979	3,566	42,788	39,222
	**4**	91,434	74,752	149,886	75,134
**in the second minute**	**1**	168,606	145,302	202,735	57,433
	**2**	156,126	138,934	167,969	29,035
	**3**	108,244	96,655	119,323	22,668
	**4**	171,408	161,984	190,000	28,016
**in the third minute**	**1**	194,584	175,610	216,997	41,387
	**2**	161,220	125,054	167,714	42,66
	**3**	99,075	96,655	132,694	36,039
	**4**	175,228	159,564	193,948	34,384
**in the fourth minute**	**1**	199,424	176,947	208,593	31,646
	**2**	154,088	111,173	165,295	54,122
	**3**	93,217	89,397	96,019	6,622
	**4**	170,134	162,748	187,708	24,96

**in the fifth minute**	**1**	211,649	193,387	229,350	35,963
	**2**	169,115	133,458	183,887	50,429
	**3**	104,169	95,255	109,390	14,135
	**4**	190,764	180,322	230,942	50,62
**in the sixth minute**	**1**	158,342	137,432	164,416	26,984
	**2**	112,828	77,172	123,016	45,844
	**3**	54,759	42,788	60,999	18,211
	**4**	139,571	126,964	148,906	21,942

Mdn, median; Q1, quartile 1; Q3, quartile 3; IQR, interquartile range
